# Electroporation-mediated gene delivery of cleavage-resistant pro–nerve growth factor causes retinal neuro- and vascular degeneration

**Published:** 2012-12-14

**Authors:** Suraporn Matragoon, Mohammed M.H. Al-Gayyar, Barbara A. Mysona, Mohammed A. Abdelsaid, Bindu A. Pillai, Kenneth E. Neet, Susan C. Fagan, Azza B. El-Remessy

**Affiliations:** 1 Program in Clinical and Experimental Therapeutics, College of Pharmacy, University of Georgia, Augusta, GA; 2 Department of Pharmacology and Toxicology, Augusta, GA; 3 Vision Discovery Institute, Georgia Health Science University, Augusta, GA; 4 Charlie Norwood Veterans Affairs Medical Center, Augusta, GA; 5 Department of Biochemistry and Molecular Biology, Rosalind Franklin University of Medicine and Science, North Chicago, IL; 6 Department of Biochemistry, Faculty of Pharmacy, University of Mansoura, Egypt

## Abstract

**Purpose:**

Neurotrophins, including nerve growth factor (NGF), are secreted by glia as a pro-form (proNGF) that is normally cleaved into the mature ligand. Increases of proNGF has been well documented in retinal neurodegenerative diseases. Since systemic overexpression of proNGF exhibits embryonic lethality, we aimed to establish a model that specifically and stably overexpresses a cleavage-resistant mutant of proNGF (proNGF123) plasmid in the retina using electroporation.

**Methods:**

Male Sprague-Dawley rats were injected intravitreally with pGFP or pGFP-proNGF123 plasmids, then electroporated with various settings for optimization. Retinal cell death and ganglion cell count were assessed by TUNEL and immunostaining with anti-Brn3. Expression of proNGF, NGF, and their receptors was examined by western blot. Retinal vascular permeability was assessed by extravasation of bovine serum albumin–fluorescein. Development of acellular capillaries was assessed by periodic acid-Schiff and hematoxylin staining.

**Results:**

Successful pGFP-proNGF123 gene delivery and expression of proNGF was demonstrated by western blot and extensive proNGF immunostaining in retina sections. Overexpression of proNGF reduced NGF expression while inducing the expression of neurotrophin receptors, including p75^NTR^ and tyrosine receptor kinase A, but not sortilin. Overexpression of proNGF resulted in ~50% reduction in ganglion cell count and fivefold increase in TUNEL-positive cells in rat retina. In addition, overexpression of proNGF induced breakdown of the blood-retina barrier evident by twofold increase in extravasation of bovine serum albumin–fluorescein after 1 week and induced the development of acellular capillaries after 4 weeks.

**Conclusions:**

Electroporation can successfully incorporate and express biologically active cleavage-resistant proNGF locally in rat retinas. Overexpression of cleavage-resistant proNGF can be a useful tool to investigate specific molecular mechanisms by which proNGF causes neurodegeneration and vascular injury in the retina.

## Introduction

Neurotrophins, including nerve growth factor (NGF), are secreted by glia as a pro-form (proNGF) that is normally cleaved into the mature ligand. Accumulation of proNGF has been detected in several neurodegenerative diseases, such as Alzheimer [1,2]. Our recent studies in models of diabetes showed significant accumulation of proNGF that was positively correlated with accelerated retinal neurodegeneration and vascular injury [3,4]. These interesting findings prompted us to examine the direct actions of proNGF accumulation in mediating retinal neuronal and vascular injury. Since the diabetic milieu is complex in nature due to the metabolic effects of hyperglycemia and multiple growth factors expressed in the eye, it is very challenging to characterize the single action of a given growth factor. Therefore, there has been a great need to develop a molecular approach to locally and stably overexpress proNGF in the rodent retina.

Since proNGF is normally cleaved by several proteases to the mature NGF, we obtained a construct of the cleavage-resistant proNGF123 that was originally developed by the Neet laboratory [5-7], conjugated it to the GFP plasmid, and pioneered its retinal overexpression using intravitreal injection followed by electroporation (ELP) as a mechanism to deliver DNA to the retinal ganglion cells (RGCs). The principle of ELP relies on the fact that electrical pulses permeate cell membranes, inducing an electrophoretic influx of DNA into the target cell [8]. Electroporation technique has been successfully used for the delivery of genes into the RGCs with minimal tissue damage. Examples include delivering different genes such as αA- and αB-crystallins [9], glial cell line–derived neurotrophic factor [8], and thioredoxin [10]. In addition, in vivo ELP was also reported in the corneal endothelium [11,12] and retinal pigment epithelial cells [13].

NGF and proNGF, both ligands of the neurotrophin receptor p75^NTR^, have opposing roles depending on the association of p75^NTR^ with its coreceptors, which are tyrosine receptor kinase A (TrkA) and sortilin. NGF and proNGF exhibit relatively low affinity when binding to either p75^NTR^ or TrkA alone [14,15]. The association of the p75^NTR^ receptor with coreceptor TrkA, however, results in a complex with high affinity for NGF, but not proNGF. TrkA kinase has intrinsic kinase activity, through which the NGF/p75^NTR^/TrkA complex activates prosurvival pathways. In contrast, proNGF, but not NGF, has a high affinity for p75^NTR^ in association with coreceptor sortilin to stimulate primarily proapoptotic events [16]. This study aimed to demonstrate the feasibility of proNGF overexpression and to characterize the actions of proNGF on the expression of its receptors, RGCs, and the integrity of the blood-retina barrier (BRB) function. We also examined the effects of overexpressing proNGF on the development of acellular capillary formation, a hallmark of retinal ischemia. Establishing such a rodent model will facilitate better characterization of proNGF accumulation in mediating retinal neuronal and vascular injury as well as the signaling events initiated by proNGF.

## Methods

### Animal preparation

All procedures with animals were performed in accordance with the Association for Research in Vision and Ophthalmology Statement for the Use of Animals in Ophthalmic and Vision Research, and the Charlie Norwood VA Medical Center Animal Care and Use Committee (protocol number 10–05–026). Three sets of Male Sprague-Dawley rats (~250 g body weight), with a total of 53 animals, from Harlan Laboratories (Indianapolis, IN), were housed in a 12 h:12 h light-dark cycle at a controlled temperature and humidity with free access to food and water.

### Plasmid preparation

Murine proNGF (980 bp) mutated at three sites (proNGF123) necessary for cleavage into mature NGF (proNGF123; 5) was kindly provided by Dr. Kenneth E. Neet, Rosalind Franklin University of Medicine and Science, North Chicago, IL. ProNGF123 was subcloned into pAcGFP1-C3 (Clontech Laboratories, Mountain View, CA) at HindIII and EcoRI sites. The construct has a strong CMV promoter that drives expression of GFP, which is used for monitoring transduction efficiency. The newly constructed plasmid was purified using EndoFree plasmid Maxi kit from Qiagen (Valencia, CA) according to manufacture protocol.

### Gene transfer into retinal ganglion cells with electroporation

Plasmids including pGFP alone or pGFP-proNGF123 were delivered to retinal cells by ELP. ELP-mediated gene delivery was performed as described previously [10] with minor modifications. Rats were anesthetized with an intraperitoneal (IP) injection of 75 mg/kg ketamine and 10 mg/kg xylazine. Five microliters of plasmid DNA were injected into the vitreous cavity with a 30 gauge needle, 0.5 mm posterior to the limbus under stereoscopic microscopy. After 10 min, the cathodal electrode was placed on the cornea, and an anodal electrode was placed at the back of the eye. Tweezertrodes (7 mm in diameter and 10mm in length) were used from BTX, San Diego, CA) catalog number is 45-0118. Electrical pulses were generated by an ECM 830 Pulse Generator (BTX). To ensure the proper delivery and even distribution of the intravitreal injection, all solutions for intravitreal injection contained 5 µg/ml Fast Green FCF (Sigma). Neomycin/polymyxin-B/bacitracin ophthalmic ointment was applied to the injected eyes and animals were allowed to recover.

### Optimization of electroporation setting using various plasmid levels

Two different electric field strengths, 6 and 12 V/cm, were tested. The pulse duration was adjusted at 100 ms and a frequency of one pulse per second. Two stimulation patterns of 5 and 10 pulses were used. After a 10 min pause, more pulses with the same parameters were delivered. Next, two different levels of plasmid (5 µg/eye) and (20 µg/eye) were compared and animals were sacrificed after 3, 7, or 21 days.

### Determination of transduction efficiency

Retinal flatmounts were fixed using 2% paraformaldehyde in PBS (137 mM NaCl, 2.7 mM KCl, 10 mM Na_2_HPO_4_, 2 mM KH_2_PO_4_, pH 7.4) and reacted with monoclonal anti-GFP antibody (Millipore, Billerica, MA), followed by Oregon-green-conjugated goat antimouse antibodies (Invitrogen, Carlsbad, CA). Retinas were counterstained with 4',6-diamidino-2-phenylindole (DAPI). Under a fluorescence microscope (Zeiss, Jena, Germany), eight areas were chosen at random (at least five positive cells were chosen in each area). The transduction efficiency was calculated as the percentage of the number of GFP-positive cells to the total number of DAPI positive cells/retinal field. Western blot (WB) analysis using anti-GFP antibody (Clontech Laboratories, Mountain View, CA) was used to detect GFP expression in retina homogenate.

### Immunolocalization studies

Optimal cutting temperature (OCT)-frozen sections (10 μm) of eyes were fixed using 2% paraformaldehyde in PBS and reacted with polyclonal proNGF (Alomone Labs, Jerusalem, Israel) or monoclonal anti-GFP antibody (Millipore) overnight followed by Oregon Green-conjugated goat anti-mouse or goat anti-rabbit antibodies (Invitrogen). Images (n=6 in each group) were collected using an AxioObserver.Z1 Microscope (Zeiss).

### Retinal protein extraction and western blot analysis

Retinas were isolated and homogenized in radioimmunoprecipitation assay (RIPA) buffer as described previously [17]. Samples (50 µg protein) were separated by sodium dodecyl sulfate polyacrylamide gel electrophoresis and electroblotted to a nitrocellulose membrane. Antibodies for proNGF and NGF (Alomone Labs Ltd), p75^NTR^, sortilin, and TrkA (Millipore) were used. Membranes were reprobed with β-actin (Sigma-Aldrich, St. Louis, MO) to confirm equal loading. The primary antibody was detected using a horseradish peroxidase (HRP)-conjugated goat antirabbit antibody (EMD, La Jolla, CA) and enhanced chemiluminescence. The films were scanned and the band intensity was quantified using densitometry software version 6.0.0 Software from alphaEaseFC (Santa Clara, CA) and expressed as relative optical density (ROD).

### Evaluation of retinal cell death

Quantitative assessment of retinal cell death was performed using immunoperoxidase staining of TUNEL-positive nuclei (ApopTag-Peroxidase, rom Millipore) in wholemount retina, as described previously by our group [4,17]. Briefly, formalin-fixed retinas were flatmounted, dehydrated in ethanol, defatted by xylenes, and rehydrated. After permeabilization with 0.5% TritonX-100, TUNEL-HRP staining with 3-amino-9-ethylcarbazole was performed following the manufacturer’s instructions. The total number of TUNEL-HRP-positive cells was counted in each retina using light microscopy. TUNEL was also performed in 10 µm OCT-frozen eye sections using the ApopTAG in situ cell death detection kit (TUNEL–fluorescein isothiocyanate), as described previously [4,17].

### Counting number of neuronal cells in the ganglion cell layer

Neuronal cells in the ganglion cell layer (GCL) were counted as described previously by our group [18]. Briefly, OCT frozen retinal sections were stained with hematoxylin and eosin for light microscopy. The nuclei in the GCL, not including nuclei in the vessels, were counted in four locations in the retina, including both sides of the optic nerve (posterior) and midretina (central) in a masked manner. Since neuronal cells in the GCL contain RGC and amacrine cells, ganglion cells were labeled using Brn3a monoclonal antibody Santa Cruz Biotechnology (Santa Cruz, CA) and total number of cells were counted using DAPI. Cells in the GCL were counted from one of end of the retina to the other end (retinal length). RGCs were identified as cells positive for Brn3a, a specific RGC marker, and DAPI positive in the GCL. Number of RGCs was normalized to retinal length (mm) in each section. For each animal, two sections were counted, one near the optic nerve and one located more peripherally. Four to six animals from each group and two fields for each location were used. Retinas were imaged using an AxioObserver.Z1 Microscope (Zeiss).

### Integrity of the blood-retinal barrier function

BRB integrity was measured as previously described by our group [4]. Plasma was assayed for fluorescein concentration using a plate reader (excitation 370 nm, emission 460 nm; Bio-Tek, Winooski, VT). A standard curve was established using bovine serum albumin (BSA)-fluorescein in normal rat serum. Through serial sectioning (10 μm) and imaging (200 μm) of retinal nonvascular areas, extravasation of BSA-fluorescence was detected.

### Isolation of retinal vasculature

The retinal vasculature was isolated as described previously [18]. Briefly, freshly enucleated eyes were fixed with 2% paraformaldehyde overnight. Retinal cups were dissected, washed in PBS, and then incubated with shaking using 3% Difco-trypsin 250 (BD Biosciences) in 25 mM Tris buffer, pH 8, at 37 °C for 2 h. Vitreous and nonvascular cells were gently removed from the vasculature. The vasculature was then soaked in several washes of 0.5% Triton X-100 to remove the neuronal retina. The transparent vasculature was laid out on slides and used for acellular capillary examination.

### Determination of degenerated (acellular) capillaries

Retinal vasculature sections were stained with periodic acid-Schiff and hematoxylin (PASH). Acellular capillary counts were quantified under microscope (400X) in a masked manner. Acellular capillaries were identified as capillary-sized blood vessel tubes with no nuclei anywhere along their length. The number of acellular capillaries was counted in eight different fields of the midretina and then averaged together, indicating the number/high power field of each image.

### Data analysis

The results were expressed as mean±standard error of the mean. Differences among experimental groups were evaluated by analysis of variance followed by the Tukey-Kramer multiple comparison test. Significance was defined as p<0.05. Based on previous analyses, sample size of 4-6 animals in each group was used to compare various parameters.

## Results

### Incorporation of pGFP-proNGF123 into the rat retina by electroporation

**Figure 1:**
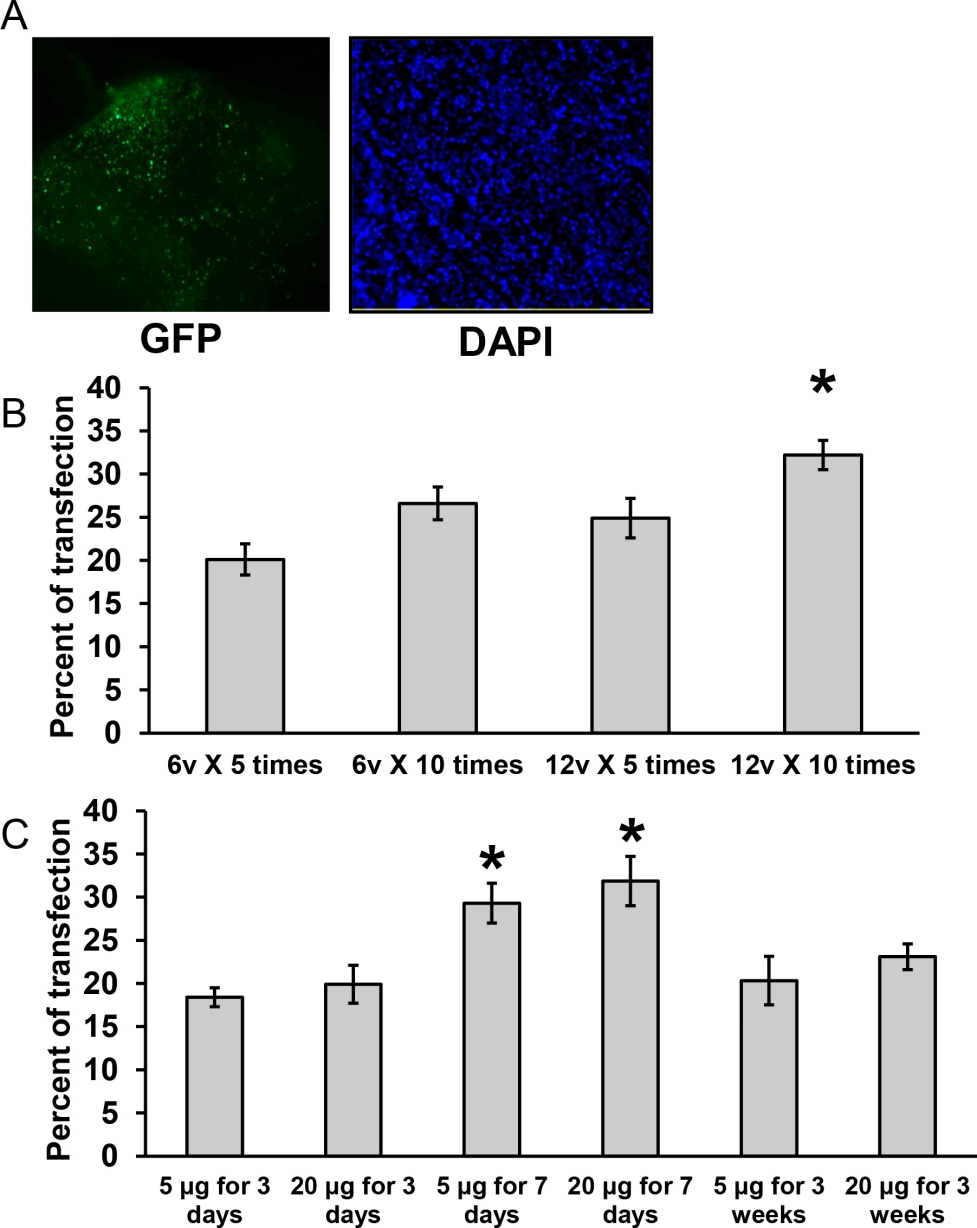
Optimization of plasmid expression in the rat retina. **A**: Representative images show the wholemount retinas stained with anti-GFP (left panel, indicating successful incorporation of the pGFP and pGFP-proNGF123 constructs in the retinas, indicated by the green fluorescence spots) and counterstained with DAPI (right panel, 400X magnification). The transduction efficiency was calculated as the percentage of the number of GFPs to the total number of nuclei in the whole retina (n=6). **B**: Statistical analysis showing changes in GFP transduction efficiency with different electroporation (ELP) settings. Four different settings were used. The transduction efficiency was measured 7 days after ELP (n=6). **C**: The statistical analysis shows changes in GFP transduction efficiency with time and plasmid concentration. Two concentrations of GFP plasmid were injected, specifically 1 µg/µl and 4 µg/µl. The transduction efficiencies were measured 3, 7, and 21 days after ELP (n=6–8). * represents significant difference as compared with the rest of the groups at p<0.05.

To further examine the actions of proNGF in mediating retinal neurodegeneration, we developed a molecular approach by incorporating the cleavage-resistant proNGF construct into GFP-conjugated plasmid that could be incorporated into the rat retina by intravitreal injection and ELP. Three days later, whole flatmounted retinas were examined under a fluorescence microscope and the transduction efficiency was calculated by measuring green fluorescence in flatmounted retinas counterstained with DAPI (Figure 1A). Various ELP settings were examined, including two electric field strengths 6 and 12 V/cm and two pulse frequencies of 5 and 10 pulses each time. As shown in Figure 1B, using field strength 12 V/cm and 10 pulses each time generated the highest signal in comparison to other groups. Therefore, we used these settings for the remainder of the study. Next, we evaluated two levels of plasmid and compared the duration of transduction. As shown in Figure 1C, although the signal was detected after all examined time points—3, 7, and 21 days—the optimum signal was detected after 7 days post injection and ELP. In addition, there was no significant difference between 5 µg plasmid/eye and 20 µg plasmid/eye (Figure 1C). Thus, 5 µg plasmid/eye for 7 days was selected for the rest of the study.

### Expression of GFP and proNGF in the rat retina

**Figure 2:**
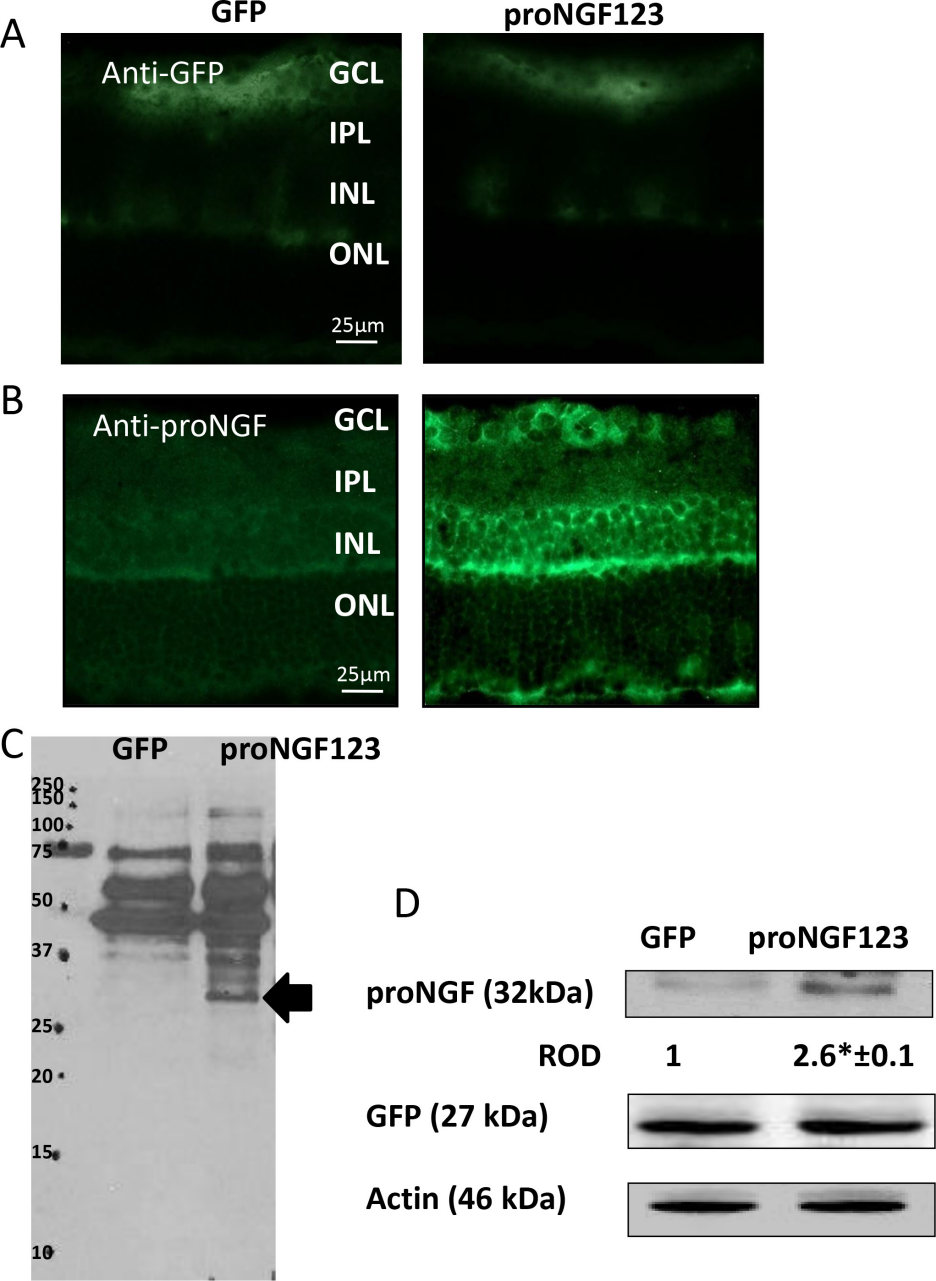
Expression pattern of proNGF in the rat retina **A**: Representative images show a similar spatial expression of GFP in retinas transduced with either pGFP or pGFP-proNGF123, indicating successful transduction and incorporation of the plasmid after electroporation (ELP; 400X magnification). **B**: Representative images show selectively increased proNGF expression in RGCs and the inner nuclear layer (INL) in rats injected with proNGF as compared with pGFP controls (400X magnification). **C**: Full length representative of western blot (WB) from vitreous samples isolated from rats injected with proNGF as compared with pGFP controls. Arrow indicates proNGF (32 kDa). **D**: WB analysis of retinal lysate showed similar GFP expression and significant increase in proNGF (32 kDa) expression in rats electroporated with proNGF123 as compared with those electroporated with pGFP (n=4). The asterisk represents a significant difference as compared with the control group at p<0.05. GCL, ganglion cell layer; IPL, inner plexiform layer; ONL, outer nuclear layer; ROD, relative optic density.

We next examined the spatial expression of GFP-expression pattern among the two groups, which showed similar green fluorescence, with the highest signal in the GCL and the inner nuclear layer (INL), and a lower signal in the outer nuclear layer (ONL; Figure 2A). In contrast, the pGFP-proNGF123 group showed enhanced proNGF expression in the RGC layer and INL as compared with pGFP controls (Figure 2B). We next attempted to characterize the expression of proNGF using WB analysis. The results showed the clear induction and release of proNGF (32 kDa) in vitreous (Figure 2C) and retinal samples transduced with proNGF123, but not from pGFP controls (Figure 2D). GFP protein was detected equivalently in all samples at its expected molecular weight (27 kDa).

### Overexpression of cleavage-resistant proNGF modulates expression of NGF, p75^NTR^ and TrkA but not sortilin

**Figure 3:**
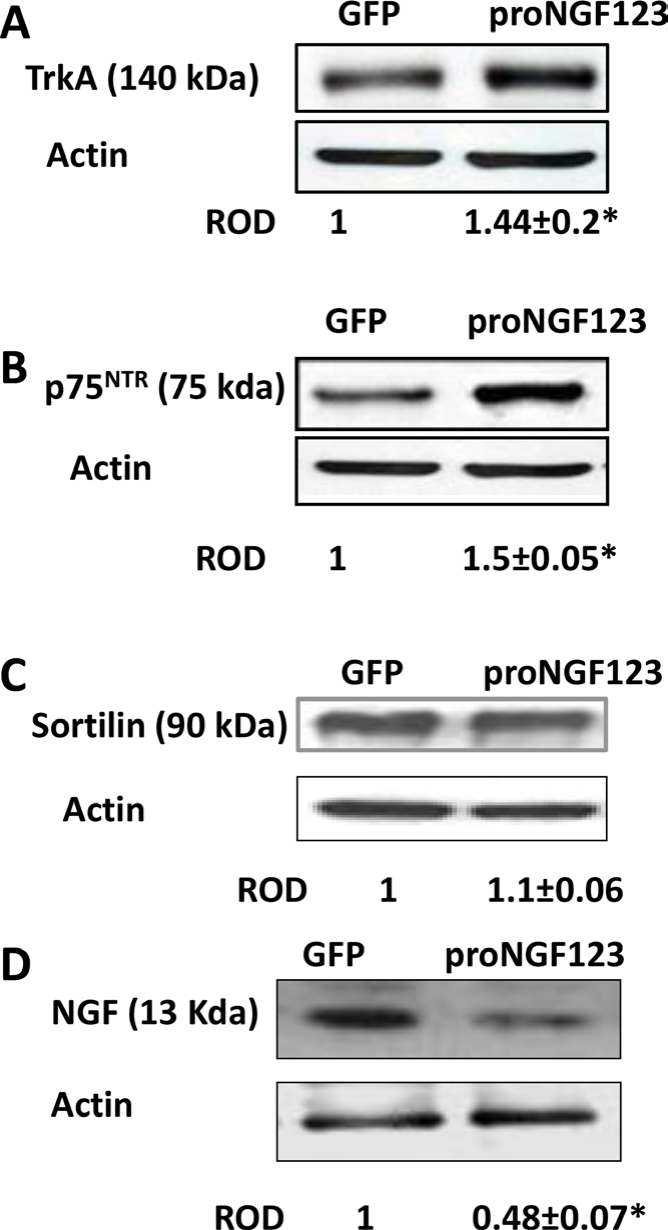
Overexpression of proNGF reduced NGF and induced expression of TrkA and p75^NTR^, but not sortilin. **A**, **B**: Western blot (WB) analysis of rat retinal lysate showed significant (1.5-fold) increase in expression of TrkA and p75^NTR^ in rats electroporated with pGFP-proNGF123 as compared with those electroporated with pGFP (n=4). **C**: WB analysis showed that overexpression of pGFP-proNGF123 in rat retinas did not affect sortilin protein expression level as compared with the control rats (n=4). **D**: WB analysis showed that overexpression of pGFP-proNGF123 significantly reduced NGF expression (n=5) compared with GFP controls. The asterisk represents significant difference as compared with control group at p<0.05. ROD, relative optical density.

Since proNGF binds to p75^NTR^, TrkA, and sortilin, we examined their expression patterns. Overexpression of pGFP-proNGF123 in rat retinas caused 1.45- and 1.5-fold increase in the expression of TrkA and p75^NTR^, respectively, as indicated by WB analysis (Figure 3A,B). However, proNGF did not modulate sortilin protein expression level as compared with the GFP-control rats (Figure 3C). Next, we assessed the effects of overexpressing proNGF on the mature ligand NGF. As shown in Figure 3D, levels of NGF (13 kDa) were significantly reduced by ~50% in retinas transduced with proNGF compared to pGFP controls.

### Overexpression of cleavage-resistant proNGF induced neuronal cell death and retinal ganglion cell loss

**Figure 4:**
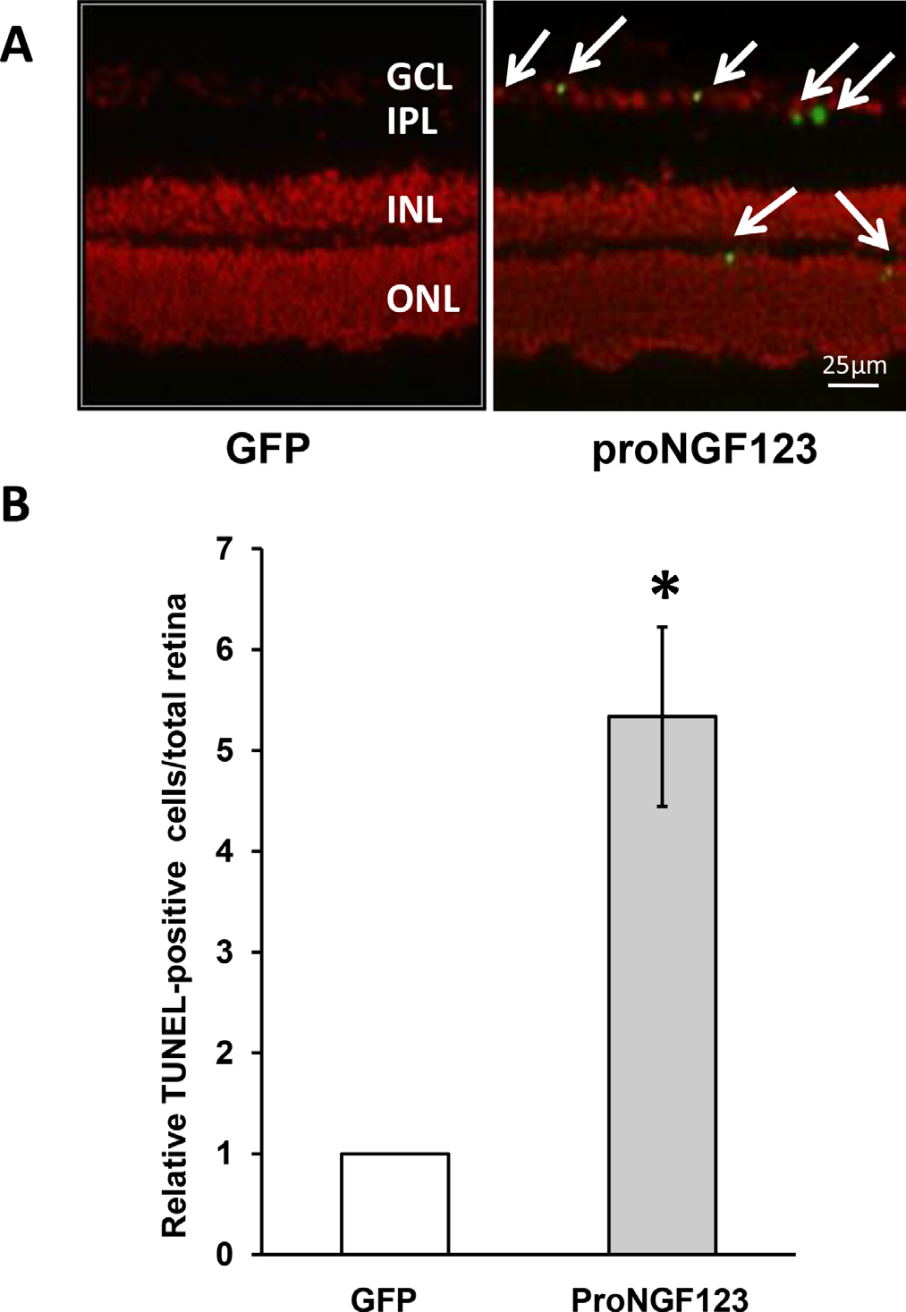
Overexpression of proNGF caused neuronal cell death **A**: Representative images of retina sections show TUNEL-fluorescein-positive cells mainly within the ganglion cell layer (GCL) and inner nuclear layer (INL) of rats injected with p-GFP-proNGF123 as compared with the pGFP control (200X magnification). **B**: Quantitative assessment of total number of TUNEL–horseradish peroxidase (HRP)-positive cells in the flatmounted retina showing 5.5-fold increase of cell death in retinas from rats injected with proNGF compared to pGFP controls (n=5–6). * represents significant difference as compared with the control group at p<0.05. IPL, inner plexiform layer; ONL, outer nuclear layer.

**Figure 5:**
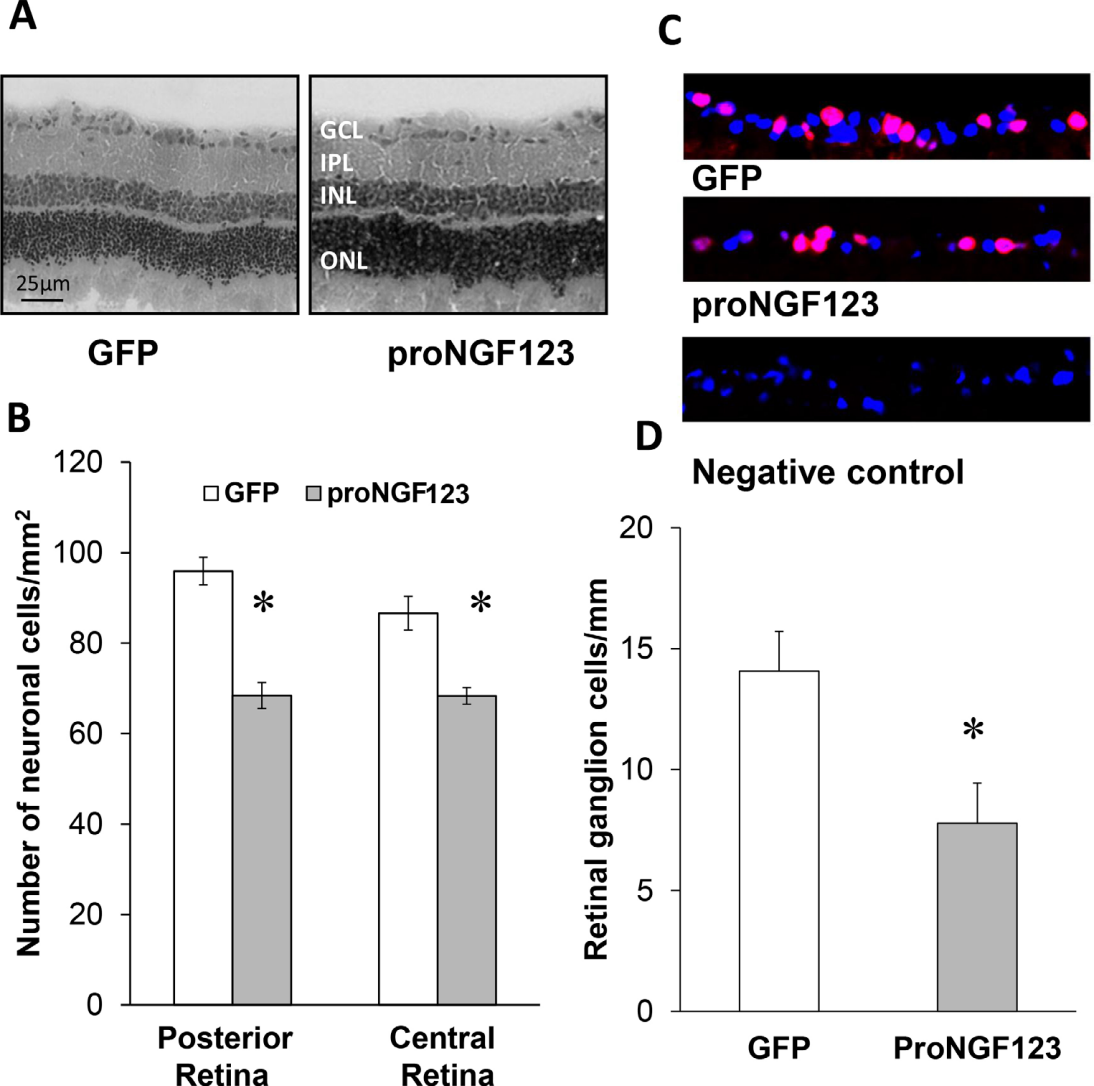
Overexpression of proNGF reduced the number of cells in the ganglion cell layer. **A**, **B**: Representative images and statistical analysis of rat retina sections stained with hematoxylin and eosin showing a reduction in number of neuronal cells in the ganglion cell layer in rats injected with p-GFP-proNGF123 as compared with pGFP controls in the central and posterior retina (n=4, 200X magnification). **C**, **D**: Representative images and statistical analysis of the number of RGC normalized to the retinal length. RGCs were counted as Brn3a-positive cells (Red) and DAPI (Blue). The results showed a significant reduction in RGC number in rats injected with p-GFP-proNGF123 as compared with pGFP controls. The asterisk represents significant difference as compared with the control group at p<0.05. IPL, inner plexiform layer; INL, inner nuclear layer; ONL, outer nuclear layer.

Our previous studies in a streptozocin (STZ)-diabetic model showed accumulation of proNGF that was positively correlated with neuronal cell death [3,4]. As shown in Figure 4A, an examination of retinal sections confirmed the detection of TUNEL-positive cells in the RGC layer. In addition, quantitative assessment of cell death by counting TUNEL-positive nuclei in flat-mounted retinal cells, showed a five-fold increase in cell death in the proNGF123 group compared to the GFP controls (Figure 4B). Moreover, as shown in Figure 5A,B, overexpression of proNGF caused neuronal cell loss, as indicated by a 25%–35% reduction in neuronal cells in the GCL in both the posterior and central retina. Since the GCL contains mixed population of RGCs and displaced amacrine cells, RGCs were labeled and counted using Brn3a antibody and normalized to retina length. As shown in Figure 5C, overexpression of proNGF caused significant and marked reduction (50%) of the RGC count compared to retinas transduced with pGFP control.

### Overexpression of cleavage-resistant proNGF caused blood-retinal barrier breakdown

**Figure 6:**
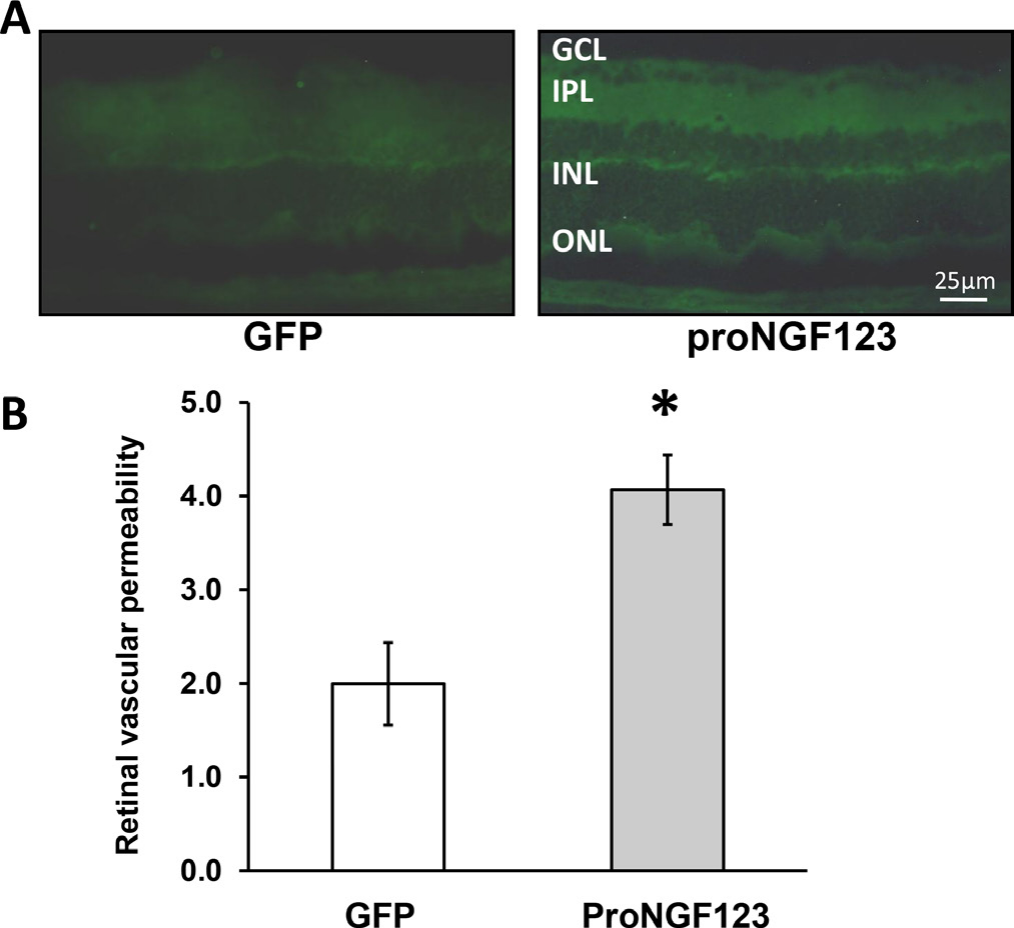
Overexpression of proNGF caused the breakdown of the blood-retina barrier. **A**, **B**: Representative image and morphometric analysis of fluorescence intensity in serial sections of rat eyes showed that retinas injected with pGFP-proNGF123 had a twofold increase in fluorescence compared with controls injected with p-GFP. The asterisk represents significant difference as compared with the control group at p<0.05. GCL, ganglion cell layer; IPL, inner plexiform layer; INL, inner nuclear layer; ONL, outer nuclear layer.

BRB integrity was assessed quantitatively using serial image analysis of retinal fluorescence intensity that was normalized to plasma fluorescence intensity for each animal. The results showed about a two-fold increase in fluorescence intensity in retinas electroporated with cleavage resistance proNGF compared with controls. Representative images showed diffuse and prominent fluorescence throughout the retinal parenchyma of retinas electroporated with p‑proNGF123, but not with the pGFP control (Figure 6).

### Overexpression of cleavage-resistant proNGF–induced retinal microvascular degeneration

**Figure 7:**
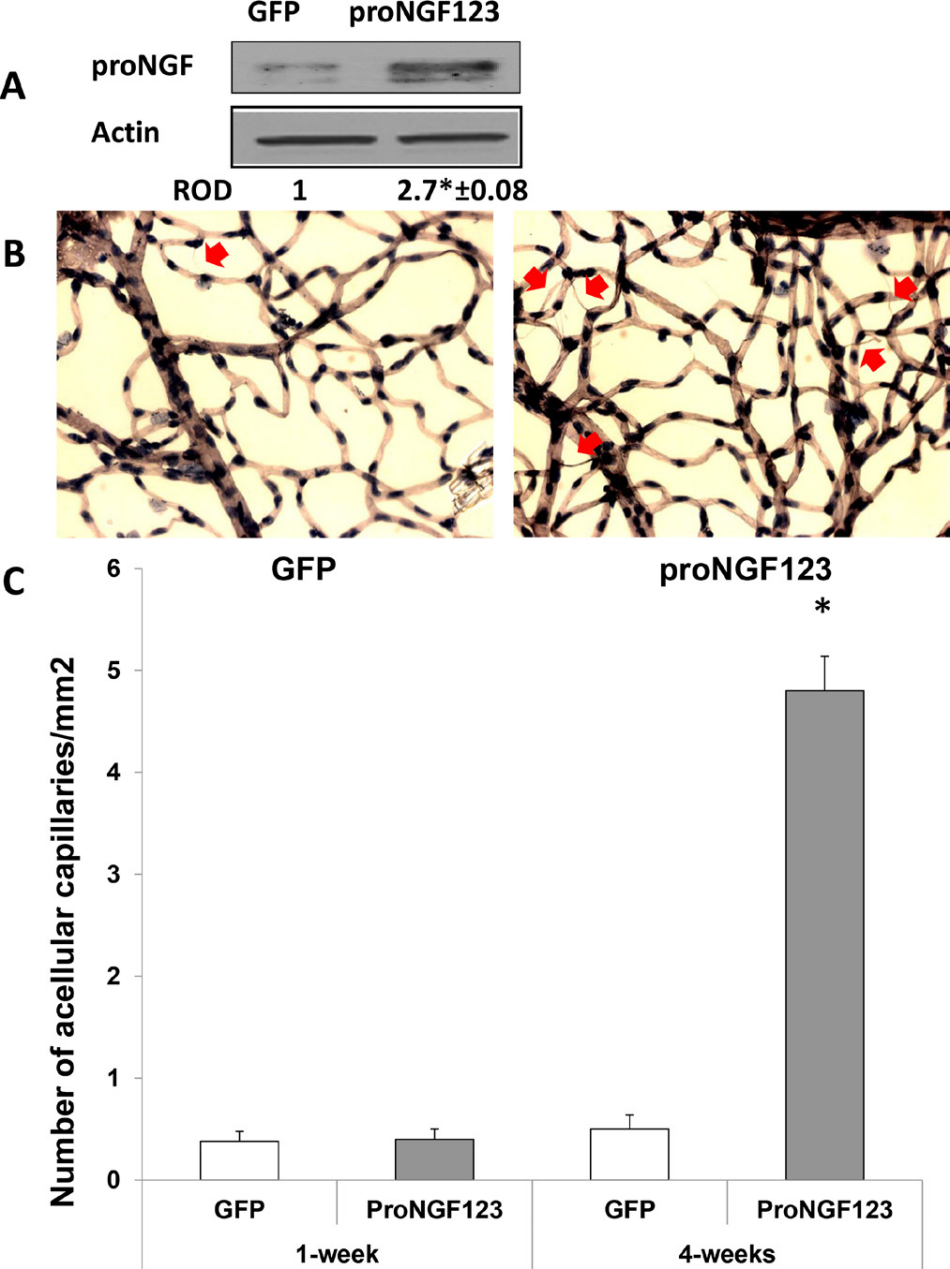
Overexpression of proNGF caused retinal microvascular degeneration. **A**: Western blot (WB) analysis of retinal lysate showed significant increase in proNGF (32 kDa) expression in rats electroporated with proNGF123 as compared with those electroporated with pGFP (n=4). The asterisk represents significant difference as compared with the control group at p<0.05. **B**: Representative images for retinal trypsin digests stained with periodic acid-Schiff and hematoxylin (PASH) showing acellular capillaries (arrows). **C**: Statistical analysis of the number of acellular capillaries showed no significant difference after 1 week of proNGF overexpression (n=4). Analyses after 4 weeks showed significant increases in the number of acellular capillaries in retinas injected with pGFP-proNGF123 when compared to the pGFP control group (n=4; * p value <0.05).

Development of acellular capillaries is a hallmark of retinal ischemia and retinopathy. We examined the effects of overexpression of proNGF on the retinal vasculature at 4 weeks post transduction. As shown in Figure 7A, expression of proNGF was maintained at similar levels seen after 1 week in retinas transduced with proNGF123, but not in pGFP controls. As shown in Figure 7B-C, retinal tryptic digests stained with PASH showed no significant microvascular degeneration after 1 week of proNGF injection. In contrast, significant increases in the number of acellular capillaries were detected in retinas transduced with proNGF123 compared to pGFP controls after 4 weeks of proNGF overexpression.

## Discussion

The main findings of the current study are as follows: 1) Successful incorporation and expression of the cleavage-resistant pGFP-proNGF123 within the GCL and INL in a rat retina via ELP; 2) overexpression of proNGF-induced expression of p75^NTR^ and TrkA but not sortilin; 3) overexpression of the proNGF decreased NGF expression, which coincided with neurodegeneration and breakdown of the BRB after 1 week, as well as microvascular degeneration after 4 weeks. We believe that this is the first study to demonstrate the actions of proNGF123 plasmid in retinal neurodegeneration/vascular injury using this molecular approach.

Neurotrophins, including NGF, are secreted by glia as a pro-form (proNGF) that is normally cleaved into the mature ligand. Our recent studies documented significant accumulation of proNGF at the expense of the mature NGF in ocular fluids and retinas from diabetic patients and rat retinas [3,4]. To characterize the role of proNGF in retinal injury apart from the complex nature of the diabetic milieu, we attempted to create a transgenic mouse that overexpresses the cleavage-resistant proNGF. A construct of the cleavage-resistant proNGF originally generated by Neet’s group [5] was engineered to be driven by the Rosa-promoter for microinjection. The results showed that systemic overexpression of cleavage-resistant proNGF exhibited embryonic lethality and halted normal growth during mid- to late embryogenesis on embryonic day 13.5 (Appendix 1). Therefore, the cleavage-resistant proNGF construct was reengineered to be conjugated to the GFP-plasmid that could be either electroporated into cultured cells in vitro or the rat retina by ELP.

The ELP technique has been widely used to selectively transfect RGCs [8,10,19,20]. In accordance with this, our results showed intense incorporation of GFP in the GCL, as well as the inner retinal layers and to a lesser extent in the outer plexiform layer (Figure 2A). These findings support the notion that while genes may initially be targeted to one type of tissue, the gene products may be delivered to a remote location [21]. The settings of ELP were optimized to achieve high transduction efficiencies without damaging the tissue by using various electrical field strengths, pulse duration, and stimulation patterns. There was no significant difference in transduction efficiency in rats injected with 5 μg/eye and those injected with 20 μg/eye. Therefore, we used the lower plasmid concentration for the remainder of the study. The optimum transduction efficiency was achieved using a single injection 5 μg/eye of plasmid with ELP settings of 12 V/cm and 10 pulses each time. Based on the transduction efficiency, we selected 7 days post injection to perform the biochemical expression of proNGF and its receptors, as well as functional assays. WB analysis showed that retinas transduced with pGFP-proNGF123 consistently expressed 2–3-fold of proNGF protein compared to retinas transduced with GFP plasmid. The expression was examined after 1 week (Figure 2) and after 4 weeks (Figure 7A). Since the proNGF123 plasmid was inserted after GFP without a stop codon in between, a fused protein that combined GFP and proNGF was expected. The results showed clear induction and release of proNGF (32 kDa) in vitreous samples (Figure 2C) and retinal lysate (Figure 2D). This band of proNGF a 32 kDa band of proNGF was not detected in GFP-transduced samples. GFP protein was detected equivalently and evenly in all samples, as expected. Interestingly, the expected fused protein of GFP-proNGF at a higher molecular weight was not detected by anti-proNGF or anti-GFP antibodies using reducing or nonreducing conditions. These findings suggest that proNGF and GFP proteins are expressed and processed to produce individual proteins from the proNGF123 plasmid. Alternatively, the fused protein cannot be recognized by commercial anti-proNGF or anti-GFP antibodies.

Neuronal cell death plays an early and critical role in the pathogenesis of several retinal neurodegenerative disorders, including glaucoma, diabetic retinopathy, uveitis, and retinal vein occlusion [3,4,22,23]. Overexpression of proNGF was found to induce significant increases in the number of TUNEL-positive cells (fivefold) in flatmounted retinas and a loss of neuronal cells in the GCL compared with GFP controls. Interestingly, while the loss of total neuronal cells in the GCL was 20%–30%, the loss of RGCs identified by Brn3a, the selective RGC marker, was 50%, suggesting the sensitivity of RGC to death signals in response to proNGF. These effects were associated with a 1.5-fold increase in neurotrophin receptor expression, including the TrkA and p75^NTR^ receptors but not the coreceptor sortilin. The observed retinal neurodegeneration in response to proNGF overexpression could be attributed to several mechanisms, including direct actions on neurons, as well as indirect paracrine effects. One of the direct actions of proNGF overexpression is reducing neurotrophic support, as indicated by marked decrease (50%) in NGF expression compared to GFP controls. Notably, these findings recapitulate the imbalance of increased proNGF/p75^NTR^ expression at the expense of NGF observed in diabetic retinas [24]. In the retina, p75^NTR^ is expressed mainly in Müller cells and RGCs [25-27]. It has been reported previously that proNGF can promote neuronal apoptosis through binding p75^NTR^ [28,29]. Upregulation of p75^NTR^ can directly activate the small GTPase protein RhoA and the stress kinase p38MAPK pathways resulting in neuronal death [30-32]. In support of this, our recent studies using a diabetic model and proNGF overexpression demonstrated neuroprotective effects, inhibiting Rho kinase activity [33] or knocking down p75^NTR^ expression [34]. In addition to the direct effects of proNGF inducing neuronal death, a proinflammatory role of proNGF in Müller glia has been well documented. We and others have demonstrated that in Müller glia cells, proNGF can stimulate tumor necrosis factor-α production in a p75^NTR^-dependent manner and cause RGC death [34,35].

Neurodegeneration was associated with breakdown of the BRB, as indicated by extravasation of BSA-fluorescein. The effects on barrier integrity could not be attributed to endothelial cell death, as our analyses showed no capillary degeneration at that time point (Figure 7C). Using the technique of injecting proNGF-plasmid followed by ELP resulted in a reproducible pattern of inducing proNGF expression that lasted for 4–6 weeks. Analyses of tryptic digest of the retina showed the development of acellular capillary formation, a hallmark of microvascular degeneration and retinopathy in retinas transduced with proNGF compared to pGFP controls (Figure 7). Recently, a mutant recombinant cleavage-resistant protein became commercially available for proNGF; this has been demonstrated to be an effective tool to examine the effects of proNGF in inducing retinal neurodegeneration [34]. However, the long-term action of a single intravitreal injection of the mutant protein remains unknown. Parallel studies were performed to examine the long-term effects (4 weeks) of mutant proNGF on microvascular degeneration. The results showed a modest effect of intravitreal mutant protein compared to saline injection (onefold). Our results suggest that plasmid incorporation should facilitate continuous expression of the proNGF and avoid the necessity for multiple injections of the mutant protein itself. As such, plasmid injection will be a more cost-effective therapy.

Notably, we attempted the same technique on the intravitreal injection of plasmid in C57Bl6 mice followed by ELP, and produced similar results [35]. However, the intravitreal injection of mice is much more challenging compared to the intravitreal injection of the rat eye. Although the current study focused on examining the detrimental effects of proNGF on retinal neuro- and vascular degeneration, we did not examine pharmacological or molecular inhibitors. Given that the eye is a confined chamber, intravitreal delivery of the plasmid alone or mixed with inhibitors will minimize the cost, the frequency of injections, and their systemic effects, as well as ensuring drug delivery to the targeted tissue. A general limitation of intraocular strategies is that results can vary depending on the precision and skill of the researcher performing the intravitreal injection. In summary, we believe that ELP -mediated gene delivery of proNGF provides a strong and reliable tool that can aid researchers to examine the actions of proNGF in mediating retinal neuronal and vascular injury.

## Appendix 1.

Embryos of both wild type and proNGF transgenic mice at embryonic day 14.5 revealed that overexpression of proNGF delayed embryogenesis. To access the data, click or select the words “Appendix 1.” This will initiate the download of a compressed (jpg) archive that contains the file.
